# The potential of biology-guided radiation therapy in thoracic cancer: A preliminary treatment planning study

**DOI:** 10.3389/fonc.2022.921473

**Published:** 2022-10-14

**Authors:** Steven N. Seyedin, Rostem Bassalow, Osama R. Mawlawi, Lehendrick M. Turner, Roshal R. Patel, Samuel R. Mazin, Oluwaseyi M. Oderinde, Yevgen Voronenko, Cody A. Wages, Peter D. Olcott, Joe Y. Chang, Peter A. Balter, James W. Welsh

**Affiliations:** ^1^ Department of Radiation Oncology, University of California, Irvine-Chao Family Comprehensive Cancer Center, Orange, CA, United States; ^2^ Northwest Medical Physics Center, Lynnwood, WA, United States; ^3^ Department of Imaging Physics, The University of Texas MD Anderson Cancer Center, Houston, TX, United States; ^4^ Department of Radiation Oncology, The University of Texas MD Anderson Cancer Center, Houston, TX, United States; ^5^ Department of Radiation Oncology, Memorial Sloan Kettering Cancer Center, New York, NY, United States; ^6^ RefleXion Medical, Hayward, CA, United States; ^7^ Department of Radiation Physics, The University of Texas MD Anderson Cancer Center, Houston, TX, United States

**Keywords:** radiation therapy, stereotactic radiotherapy, thoracic cancer, BgRT, PET guidance

## Abstract

**Purpose:**

We investigated the feasibility of biology-guided radiotherapy (BgRT), a technique that utilizes real-time positron emission imaging to minimize tumor motion uncertainties, to spare nearby organs at risk.

**Methods:**

Volumetric modulated arc therapy (VMAT), intensity-modulated proton (IMPT) therapy, and BgRT plans were created for a paratracheal node recurrence (case 1; 60 Gy in 10 fractions) and a primary peripheral left upper lobe adenocarcinoma (case 2; 50 Gy in four fractions).

**Results:**

For case 1, BgRT produced lower bronchus V40 values compared to VMAT and IMPT. For case 2, total lung V20 was lower in the BgRT case compared to VMAT and IMPT.

**Conclusions:**

BgRT has the potential to reduce the radiation dose to proximal critical structures but requires further detailed investigation.

## Introduction

In the last years, the role of stereotactic ablative radiation therapy (SABR) has expanded dramatically with clinical trials demonstrating an overall survival benefit of SABR for oligometastatic disease and early-stage lung cancer (1–3). One of the major limitations to expanding SABR to other systemic sites is the anatomic proximity of many tumors to critical structures that are particularly sensitive to radiation effects and severe toxicities associated with respiratory-induced tumor motion during SABR delivery (4). With improvements in image guidance radiotherapy, recent advances in radiation precision to reduce toxicity risk have included MR-guided radiation therapy with an increase in soft tissue visualization as well as real-time adaptive CT imaging (5, 6).

One approach to reducing surrounding normal tissue dose during SABR is to increase the precision of radiation therapy delivery. RefleXion Medical (Hayward, CA) aims to achieve this by utilizing outgoing tumor positron emission tomography (PET) imaging data to deliver a tracked dose to a moving target during the normal breathing cycle, a concept called biology-guided radiotherapy (BgRT). This may reduce the dose to sensitive structures without the need for additional motion management techniques. The first BgRT machine, known as RefleXion X1^®^, is FDA cleared for intensity-modulated radiotherapy (IMRT) and SABR, while the BgRT component is available for investigational use.

In addition, the role of the biological signature from PET images in characterizing oncological diseases (directed personalized medicine) cannot be overemphasized. Studies have shown that PET images plays a crucial role in generating robust clinical trial data to support response-adapted treatment, predict treatment outcome, and enhance tumor staging (7, 8).

In this study, we investigated the feasibility of the BgRT dose to various tumor-adjacent structures by performing a preliminary dosimetric comparison to two other modern radiation therapy techniques, volumetric modulated arc therapy (VMAT) and intensity-modulated proton therapy (IMPT), for two challenging thoracic tumors.

## Methods and materials

### About the RefleXion X1 system

#### RefleXion X1 radiotherapy machine

**Figure 1:**
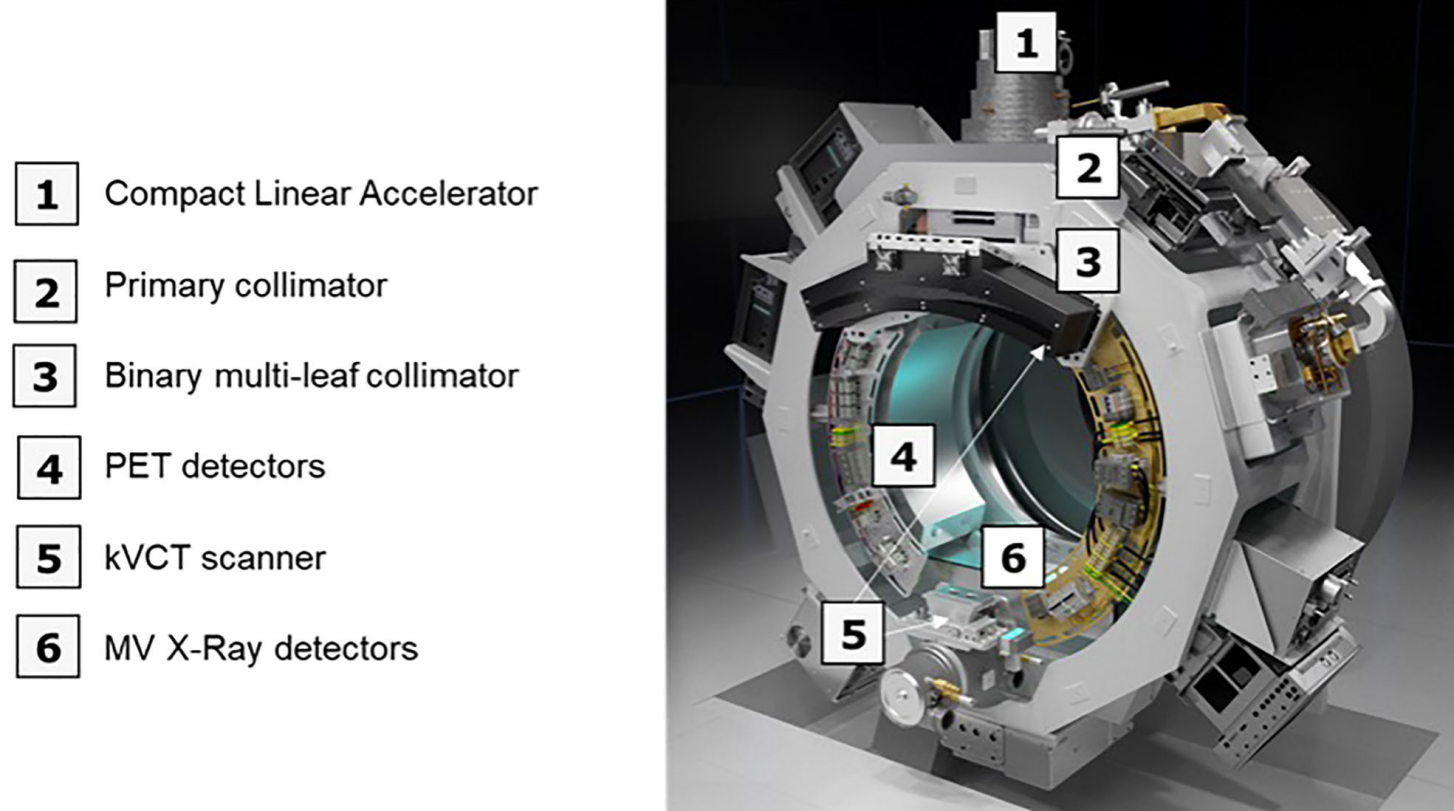
An overview of the X1 radiotherapy system showing the major components (without the couch component).

The RefleXion X1 radiotherapy machine is a linear accelerator (linac) architecture with an integrated PET, an MV detector, and a 16-slice fan beam kVCT subsystem, all mounted on a 60 RPM rotatable slip-ring gantry (**Figure 1**) (9). The linac component produces a 6-MV (flattening filter free) beam with a nominal dose rate of 850 cGy/min. The X1 has a high-speed binary multi-leaf collimator (MLC) with 64 leaves, each leaf having a 6.25-mm width at the isocenter to achieve a highly conformal treatment delivery. The PET subsystem of the X1 machine is designed to acquire PET emissions, which is useful for creating BgRT plans and guiding the beamlet delivery guidance in real time. It has two symmetrically opposed 90°C arcs of PET detectors with 64 scintillation multi-pixel counter (MPPC) modules on each arc. Animations illustrating the technology and machine design are available at the RefleXion website (10). For treatment delivery on the X1 machine, the system leverages on the couch which operates in 5 degrees of freedom and translates (in the IEC-Y direction) the patient through the therapy plane to deliver the therapeutic beam to the whole tumor length and also mitigate interplay effects.

#### BgRT contouring

In standard treatment planning for respiratory induced tumor, the internal target volume (ITV) is contoured based on the tumor/gross tumor volume (GTV) motion extent provided by the 4DCT images during the simulation CT acquisition.

**Figure 2:**
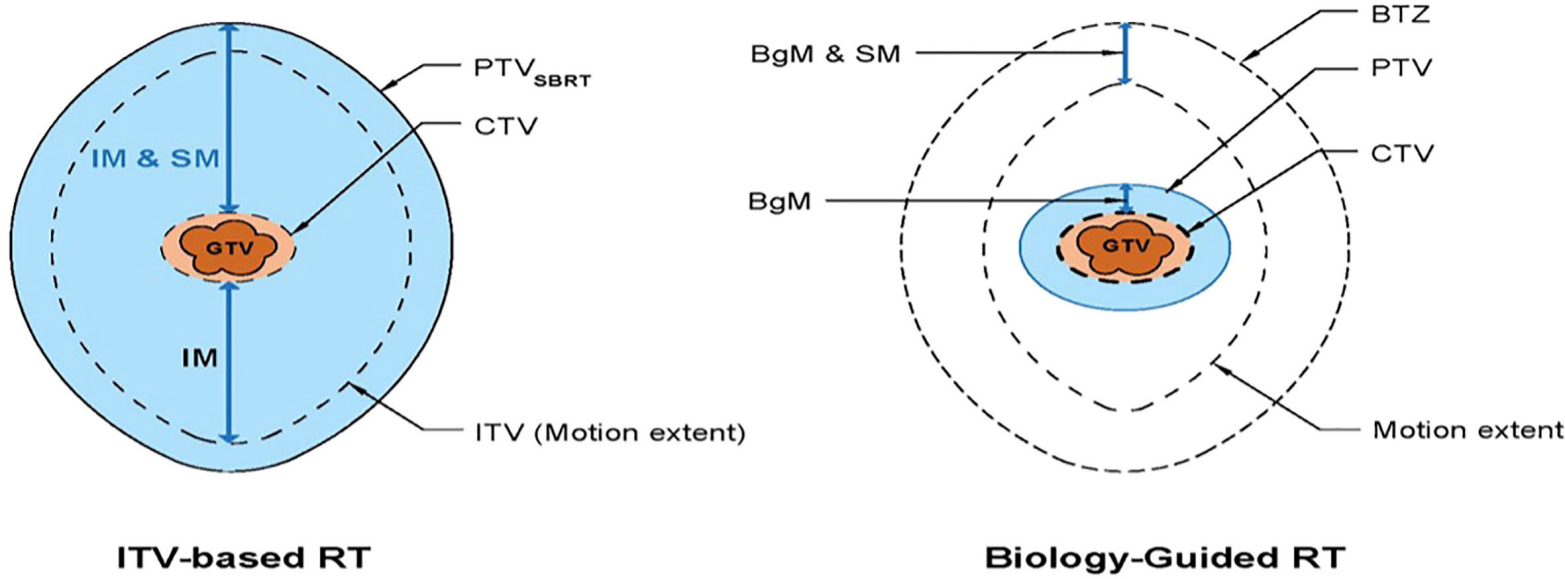
Schematic diagram showing the standard RT volumes (left) versus the BgRT volumes (8). IM, internal margin; SM, setup margin; BgM, biology-guided margin; BTZ, biology-tracking zone.

In contrast, BgRT planning uses a single-phase GTV to create a PTV from applying a biology-guidance margin (BgM) which accounts for intrinsic biologic guidance localization uncertainties to the GTV (**Figure 2** **)**. A second volume generated is the biology-tracking zone (BTZ) which encompasses the motion extent of the GTV with addition of the BgM. The BTZ is not a treatment volume but the limiting region where the treatment dose would be delivered. It means that the PET signal from outside the BTZ would not be a useful information to guide the therapeutic beamlet delivery in real time. Due to the PET biological signature, a 3-mm BgM was chosen instead to account for biological localization uncertainties in the BgRT workflow and algorithm. In addition, a 4-mm isotropic expansion was included to account for the BTZ dose delivery region extent. These expansions were selected due to the spatial resolution of the RefleXion X1 PET detectors (11). The BgRT planning process consists of first defining the target coverage goals and organ-at-risk (OAR) constraints and subsequently calculating a fluence mapping from a planning PET image to achieve the desired dose objectives using the cost function optimization process.

#### BgRT treatment planning system

BgRT delivers a radiotherapy plan to the tumor envelope using the annihilated photons emanating from the PET avidity tumor. It allows radiation dose delivery based on the collection and processing of PET data from a radiotracer that is injected into a patient on the day of treatment. In this way, BgRT uniquely utilizes radiotracer uptake as a biological beacon for targeting, tracking, and adjusting dose delivery in real time to account for target motion. Before treatment, patient PET data are collected to evaluate the patient’s candidacy for BgRT treatment. For ^18^F-FDG, the acceptable threshold for activity concentration (AC) and the normalized tumor signal (NTS) are 5 kBq/cc and 2.7, respectively. However, these metrics may change for non-FDG radiotracers.

The BgRT treatment planning system (TPS) is built to use the PET data to optimize the treatment plans. The first step in the treatment planning process is to import the CT simulation and CT-defined RT structures to the BgRT TPS. Then, the physician will define the prescribed dose objectives, and the patient is approved for PET data acquisition procedure on the X1 machine. Post PET data acquisition, the BgRT TPS algorithm will then use the acquired PET data to create a fluence map that will satisfy the prescribed dose objectives. In this study, the PET data used for BgRT planning were acquired on a third-party diagnostic static ring PET system (PET data from the institutional archive) and extrapolated to a rotating dual arc PET system on the X1 machine.

#### BgRT plan delivery

During BgRT delivery, the machine utilizes rapidly acquired “limited time sampled (LTS)” PET images to guide the beam using the mapping calculated during BgRT planning. The LTS imaging data include 500 ms of data acquisition but are updated every 100 ms in a sliding-window scheme. The average latency is around 400 ms and is compensated by adding a BgRT-related margin at the time of planning. This allows for tracked delivery to the target in real time as it moves within the BTZ. The updated LTS imaging data at the 100-ms interval is used to calculate a partial fluence, which is segmented into machine instruction to deliver the fluence until the whole planned dose is delivered to the PET-avid tumor. BgRT accounts for various treatment- and tumor-motion-related uncertainties, potentially allowing its safe use to treat tumors that are both close to critical structures and mobile (i.e., lung malignancies). A prior iteration of the BgRT algorithm has been published (9, 12).

### Patients and dose rationale

Because 4D-PET/CT data were required in order to create BgRT plans at the time this study began, we accessed an archive of patients with available ^18^F-FDG-PET/CT data without iodine contrast collected through approved institutional review board (IRB 2008-0853). From this set, two patients with lung tumors amenable to SABR were selected for dosimetric analysis. All data were appropriately anonymized.

The first patient was a 76-year-old woman who had been treated twice with VMAT (once to 50 Gy in 10 fractions and the second time to 60 Gy in 10 fractions) for a right middle lobe bronchoalveolar carcinoma. Approximately 6 months after the second treatment, a recurrence developed in a right lower paratracheal node. This node was encompassed in the GTV, which was expanded to create the planning target volume (PTV), as described below. Plans for BgRT, VMAT, and IMPT were created with the goal of administering 60 Gy in 10 fractions (BED 96; α/β =10) to at least 95% of the PTV while attempting to minimize the dose to the aorta, bronchus, chest wall, and esophagus. This prescription was considered to be safe for using SABR to treat centrally located tumors and was recently confirmed by initial phase I results of RTOG 0813 data finding 12 Gy per fraction to be the maximal tolerated dose (13). In addition, our institution had published data validating the efficacy of treating central tumors in 10 fractions when unable to meet normal structure constraints for four fraction plans (14). This case was chosen for the difficulty in meeting normal-tissue dose constraints because of the retreatment and the dose overlap within critical structures.

The second treatment-plan comparison was for a 75-year-old woman with a newly diagnosed 2.5 cm × 2.5 cm lung adenocarcinoma in the left upper lobe, with close proximity to the chest wall. BgRT, VMAT, and IMPT plans were created to deliver 50 Gy in four fractions to at least 95% of the PTV while attempting to minimize the chest wall V30 and the dose to the left lung. This dose and fractionation produces a BED of 112.5, and BED ≥100 has been retrospectively found to produce an approximate 90% local control rate at 3 years (15).

Initial 4D positron emission tomography/computerized tomography (PET/CT) data were obtained with a GE Healthcare Discovery PET/CT 690.

### Treatment planning parameters for all plans

Because the patients selected from an IRB imaging study, radiation planning was performed from the 4D-CT imaging previously obtained as part of the study described above rather than a standard CT simulation.

VMAT and IMPT plans were created using commercial treatment planning systems. BgRT plans were created using research treatment planning software (v2017, RefleXion Medical, Hayward, CA). The same planning goals and OAR constraints were used for planning across all modalities (BgRT, VMAT, IMPT). All three modalities for both patients were normalized such that PTV D_95_, the fraction of the prescription dose covering 95% of the PTV volume, was equal to the prescribed dose (D_rx_) in order to facilitate comparison of critical structure doses for all three modalities. A recent retrospective analysis of early-stage lung cancers treated with SABR noted higher rates of local control when PTV D_95_ BED_10_ was greater than 86 Gy, making PTV D_95_ an ideal normalization metric (16). D_1_ (fraction of the prescription dose that covers 1% of the PTV) was calculated for both cases to represent the maximum PTV dose, which was then divided by the prescribed dose to reflect differences in treatment heterogeneity.

For VMAT and IMPT plans, a GTV was contoured on a single phase from the 4DCT scan. No other 4D imaging was used to generate the treatment volume. For case 1, 6-mm isotropic expansions to generate the PTV from the GTV were chosen as mediastinal recurrences demonstrate minimal motion within 7 mm (17). Additionally, due to prior radiation treatment, smaller margins were chosen to minimize treatment volumes and prior dose overlap. For case 2, 8-mm isotropic expansion to generate the PTV from the GTV was utilized as upper respiratory tumor display minimal motion (18).

#### VMAT planning

Volumetric modulation was achieved by using two dynamic arcs per patient. The arc arrangement for case 1 consisted of a total of 356° geometry starting at 182° and ending at 178°. The arc arrangement for case 2 consisted of a total of 184° geometry starting at 352° and ending at 176°. Collimation per arc was chosen to maximize blocking adjacent structures while providing adequate treatment to targets.

#### IMPT planning

Scanning-beam IMPT plans were created by using an inverse-planning format and single-field optimization technique, meaning that each beam individually covers the target. Appropriately weighted objectives were used to maximize coverage and conformality while minimizing hot spots. Care was taken regarding beam selection to reduce exposure to normal tissues, particularly critical structures distal to the target.

#### BgRT planning

The BgRT plan used the same GTV as the VMAT and IMPT plan. However, because the BgRT can deliver a tracked dose of radiation to a moving target, the range of motion of the target does not need to be included in the PTV expansion.

## Results

**Figure 3:**
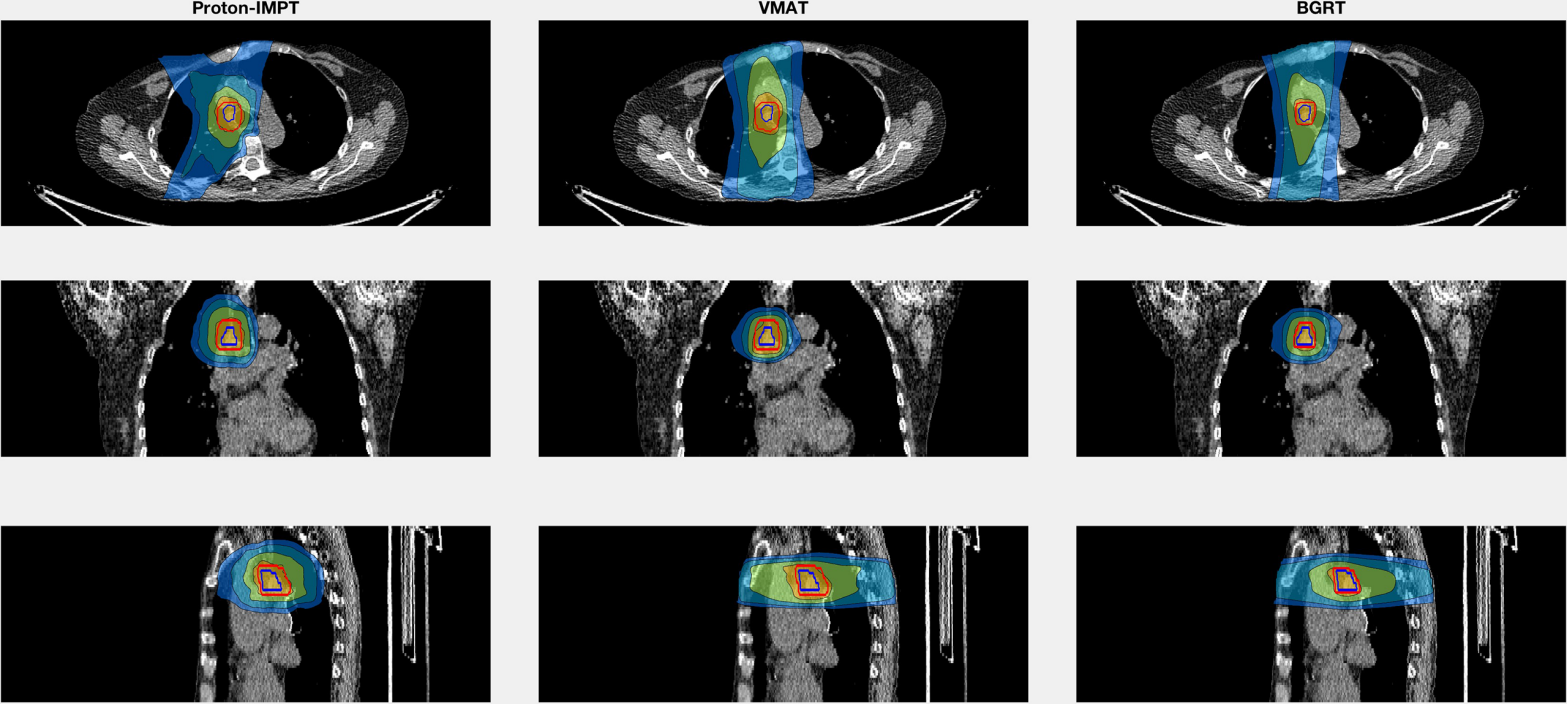
Isodose line images for case 1, involving a right lower paratracheal node recurrence after two VMAT regimens for a right middle lobe bronchoalveolar carcinoma. Isodose lines depicted are 10 Gy (blue), 20 Gy (teal), 40 Gy (green), and 60 Gy (yellow). GTV is contoured in blue. PTV displayed in red (6 mm GTV expansion for IMPT and VMAT and 3 mm for BgRT).

**Figure 4:**
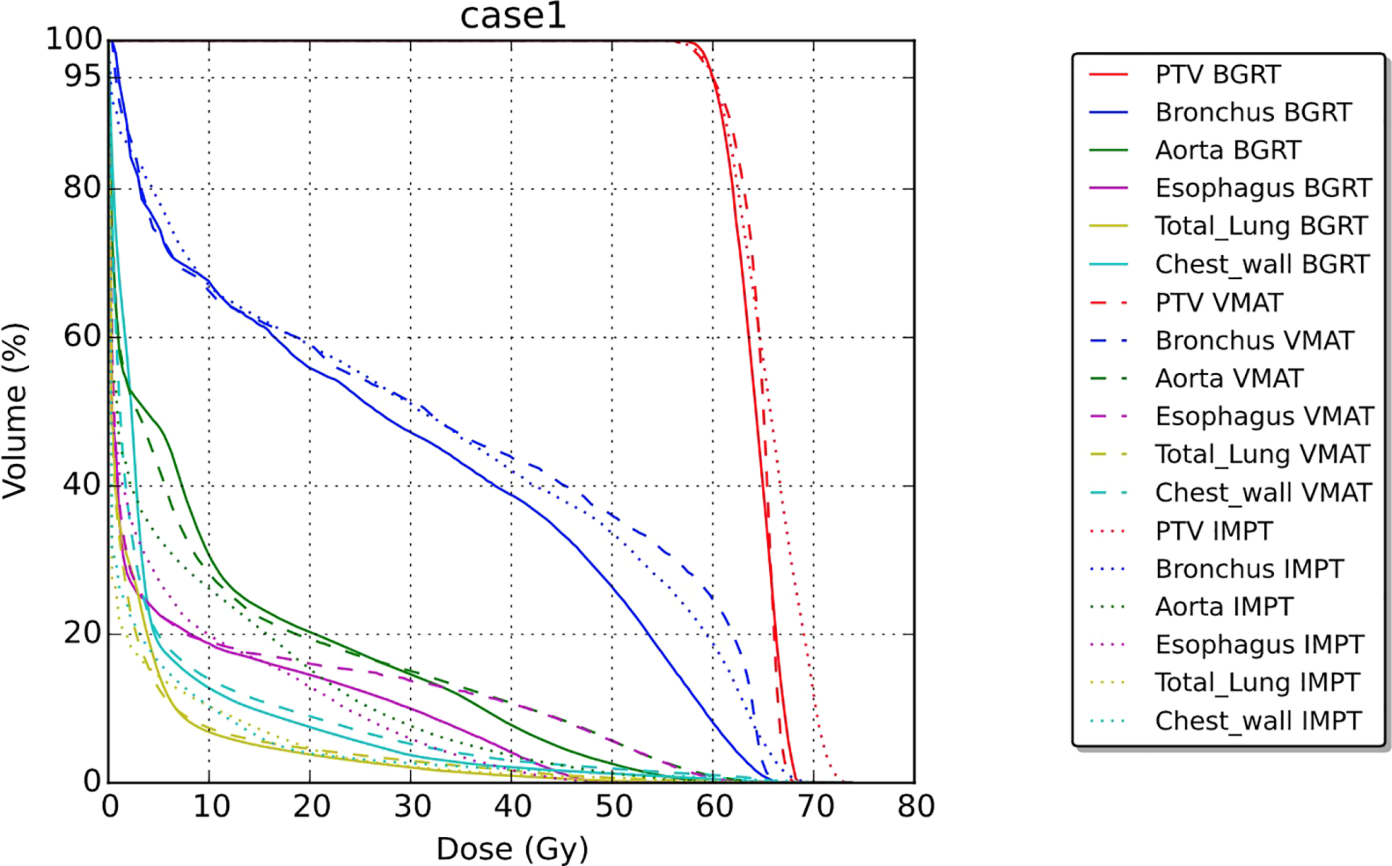
Dose–volume histogram for case 1.

**Table 1** summarizes results for the two patients. In terms of doses to critical structures for the first patient, BgRT delivered comparable maximum point doses of 1 cc of the aorta (BgRT 56.4 Gy, VMAT 61.2 Gy, and IMPT 53.9 Gy) and 5 cc of the aorta (47.9 Gy BgRT, 54.2 Gy VMAT, and 40.6 Gy IMPT). BgRT decreased bronchus V40 values (38.7%) compared to 43.7% for VMAT and 41.9% for IMPT, and bronchus D5cc doses (46.5 Gy BgRT) compared to 54.6 Gy VMAT and 51.3 Gy IMPT. BgRT esophageal V40 were lower (4.0%) than VMAT (10.6%) but higher than IMPT (1.7%), while the D5cc values were similar (1.8 Gy) to both VMAT (2.2 Gy) and IMPT (3.7 Gy). Similar degrees of treatment inhomogeneity (D_1_/D_rx_) were present in the BgRT (114.3%) and VMAT (113.1%) plans, but the IMPT plan was higher (121.2%). CT images with isodose lines and the corresponding DVH data are shown in **Figures 3**, **4**.

**Table 1:** Dose–volume statistics for the two clinical cases.

Case 1	BgRT	VMAT	IMPT
D1/Drx, % Bronchus V40, % (cc)	114.3 38.7 (6.0)	113.1 43.7 (6.7)	121.2 41.9 (6.5)
Bronchus D1cc, Gy	61.2	64.9	64.9
Bronchus D5cc, Gy Aorta V40, % (cc) Aorta D1cc, Gy	46.5 7.6 (10.8) 56.4	54.6 10.7 (15.2) 61.2	51.3 3.5 (5.0) 53.9
Aorta D5cc, Gy	47.9	54.2	40.6
Esophagus V40, % (cc)	4.0% (0.6)	10.6 (1.7)	1.7 (0.3)
Esophagus D1cc, Gy Esophagus D5cc, Gy	36.9 1.8	49.4 2.2	29.9 3.7
Mean lung, Gy Lung V20, % Chest wall V30, %	5.9 3.8 3.7	6.1 4.5 5.2	4.5 4.5 2.6
**Case 2**	**BgRT**	**VMAT**	**IMPT**
D1/Drx, % Chest wall V30, % (cc) Mean lung, Gy	115.0 1.4 (27.8) 4.7	108.7 3.6 (71.1) 5.7	122.6 2.1 (40.6) 4.6
Lung V20, %(cc)	7.5 (312.8)	10.8 (449.0)	9.3 (385.1)

**Figure 5:**
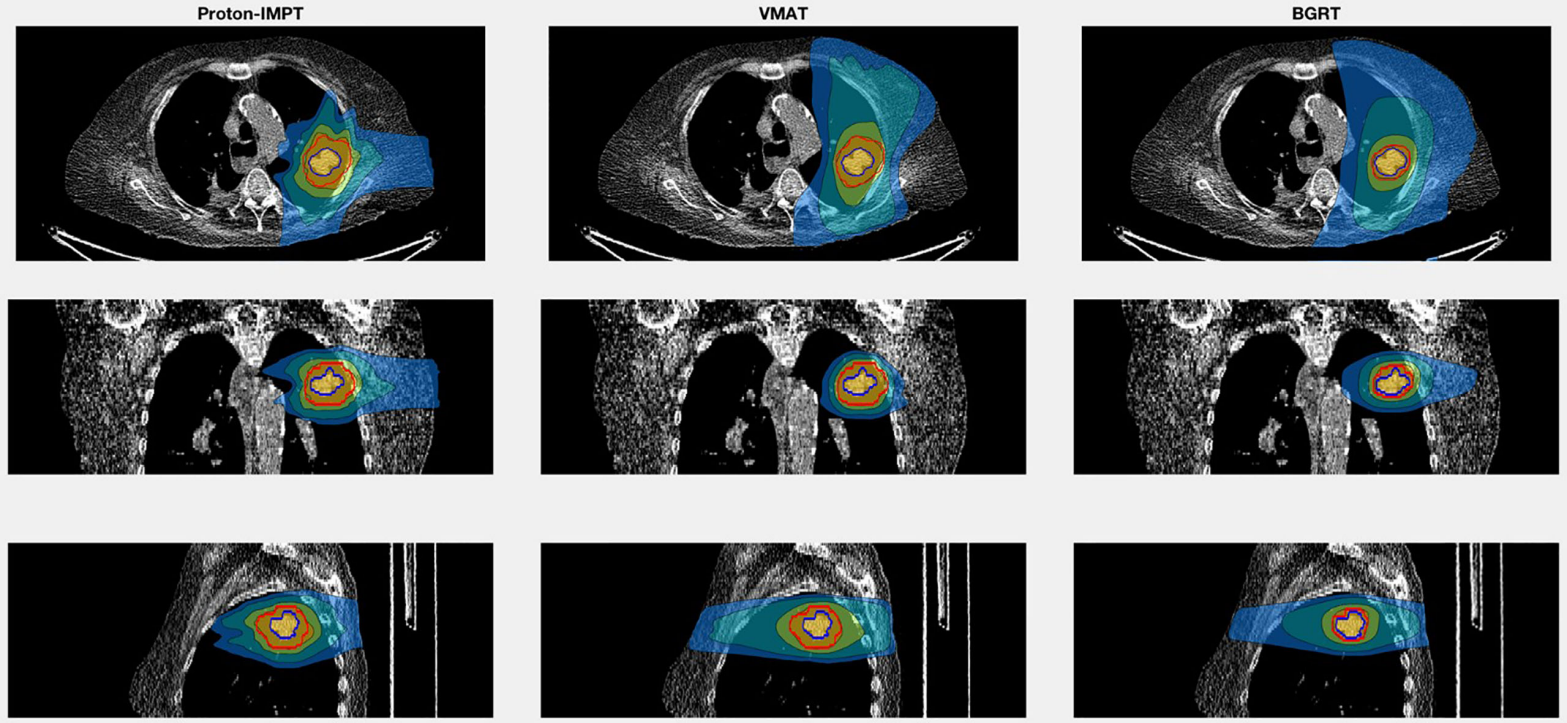
Isodose line images for case 2, involving a newly diagnosed adenocarcinoma in the upper lobe of the left lung. Isodose lines depicted are 10 Gy (blue), 20 Gy (teal), 40 Gy (green), and 50 Gy (yellow). GTV is contoured in blue. PTV displayed in red (8 mm GTV expansion for IMPT and VMAT and 3 mm for BgRT).

**Figure 6:**
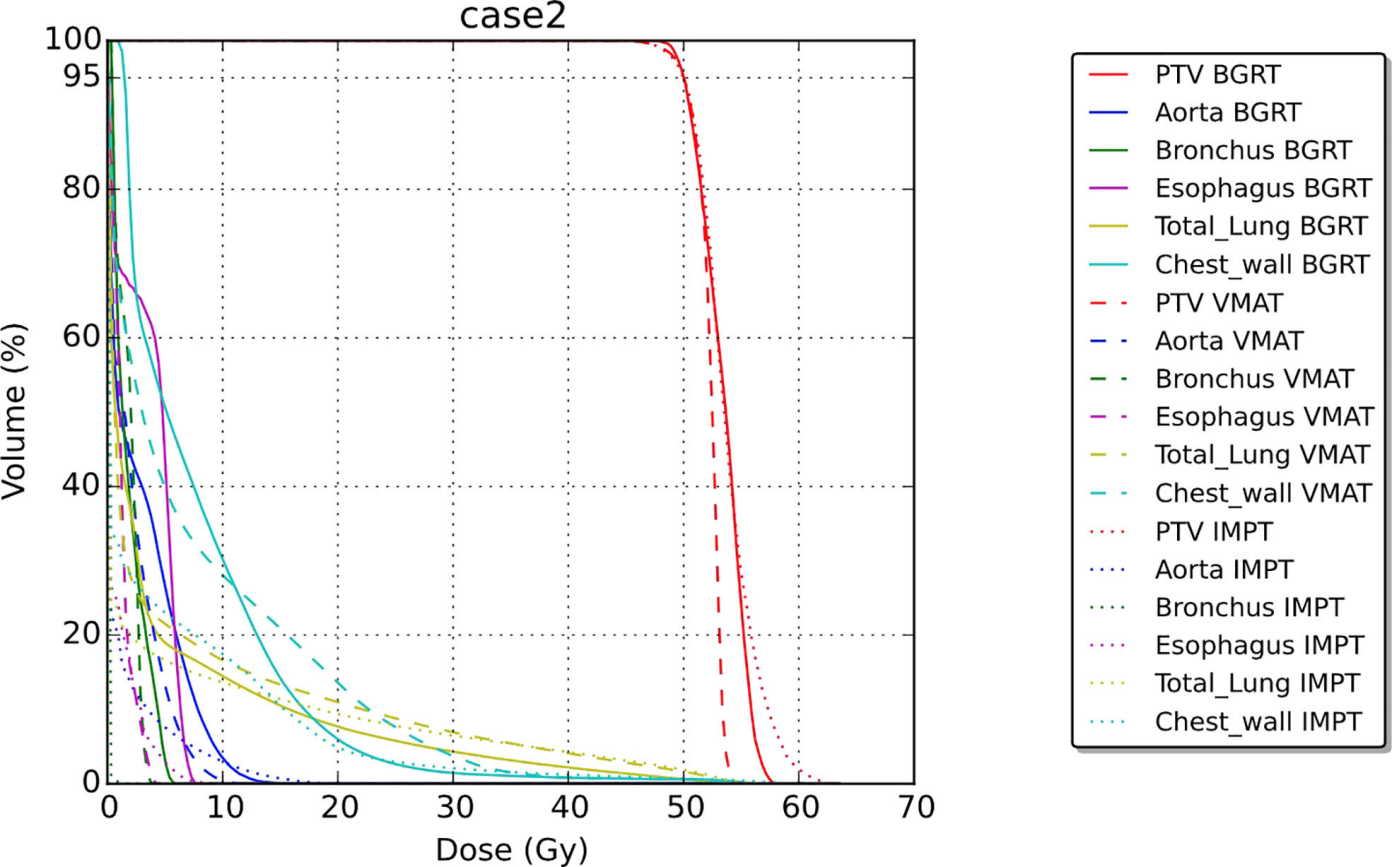
Dose–volume histogram for case 2.

For the second patient, involving SABR to the chest wall, the BgRT chest wall V30 (1.4%) was less than those of VMAT (3.6%) and IMPT (2.1%). Total lung V20 was also lower in BgRT (7.5%) compared to VMAT (10.8%) and IMPT (9.3%). The D_1_/D_rx_ for the BgRT, VMAT, and IMPT plans were 114.9%, 108.6%, and 122.5%, respectively. CT images with isodose lines and the corresponding DVH data are shown in **Figures 5**, **6**.

## Discussion

This preliminary treatment planning report investigated the feasibility of using BgRT to deliver a therapeutic beam to adjacent OAR and compare the dosimetric performance to VMAT or IMPT for the treatment of challenging thoracic tumors. Although BgRT uses traditional linac technology with an integrated PET subsystem, it represents an advancement in its consideration of both tumor motion and treatment-related uncertainties.

Our first case involved a paratracheal lymph node relapse after VMAT for two primary bronchoalveolar carcinomas in the right middle lobe. A retrospective evaluation of the composite dose to the aorta for 35 patients who received radiation as retreatment for NSCLC showed that composite doses of >120 Gy to 1 cc of the aorta correlated with high-grade aortic toxicity (19). The location of the primary (previously treated) lesions near the aorta in our first case (**Figure 3**) prompted us to evaluate the aorta dose. We found that BgRT and IMPT would produce similar maximum doses to 1 cc of the aorta and thus equivalent 1 cc composite doses. Additionally, a lower bronchus V_40_ (11% lower in the BgRT plan than VMAT and 8% lower than in the IMPT plan) could reduce the risk of radiation-induced bronchial necrosis (20, 21).

In the second case, we investigated radiation exposure to the total lung and chest wall for the treatment of an upper lobe tumor, a situation commonly encountered in the clinic. A previous investigation of SABR for thoracic tumors noted that a chest wall V30 greater than 30 cc was associated with higher rates of chest wall toxicity (22). Of the three treatment techniques examined here, the BgRT plans had lower V30 values (27.8 cc) compared to VMAT and IMPT (71.1 cc and 40.6 cc, respectively). BgRT had the lowest V20 for the total lung (7.5%) compared to IMPT (9.3%) or VMAT (10.8%), which is notable because higher lung V20 values are associated with increased risk of radiation pneumonitis (23). The ability of BgRT to reduce exposure of the chest wall and lung could reduce the risk of acute and chronic radiation-induced toxicity.

The primary goal of this study is to explore the feasibility and potential of BgRT to reduce nearby organ dose. Nevertheless, limitations are present in this dosimetric comparison study. Because we compared treatment plans for only two patients, no statistical comparisons were possible. Minor dose reductions to nearby critical structure obtained by BgRT may not translate to a clinical benefit. BgRT targets metabolically active tissue emitting a positron from ^18^F tagged to a modified glucose unit; however, elevated glucose metabolism occurs in a variety of conditions other than cancer (24). This could lead to mistreatment of falsely positive PET avid non-malignant conditions if not accounted. Finally, smaller PTV expansion margins for BgRT plans compared to IMPT and VMAT plans produce a smaller PTV volume which reduces the dose to nearby structures. Further experimental work is required to validate the safety of the 3-mm GTV expansion margins used to create the PTV for the BgRT plans. However, the purpose of the study was to explore the dosimetric reduction potential of BgRT due to its ability to track tumor motion and deliver radiation using PET signaling in real time, which prompted the use of smaller margins than conventional IMRT or VMAT planning. Finally, the authors acknowledge that although our model attempts to limit the doses to critical structures in the VMAT and IMPT plans, these dose distributions may vary slightly in 4D gated plans, which is currently under investigation.

Because BgRT utilizes positron emissions to target radiation, logistical obstacles remain when integrating the technology into clinical practice. Various steps in the workflow including imaging, dose evaluation, and BgRT delivery require further elucidation. Although radiation-induced inflammation tends to peak at several weeks after treatment, high avidity in surrounding areas that are inflamed after single-fraction high-dose radiation certainly poses an obstacle for use of BgRT (25). Because patients undergoing BgRT would require daily injections of a positron-emitting radioactive tracer, BgRT would ideally be suited for patients who need high-dose hypofractionated SABR treatment. The use of SABR has increased over the past decade especially in the setting of metastatic disease with or without immunotherapy (26–28). BgRT could be applied in the oligometastatic setting, improving the workflow and allowing SABR on a greater number of isocenters. Because BgRT may be able to target the GTV in real time, another possible approach to integrating it into clinical practice would be for weekly boosts to IMRT.

In summary, because BgRT can provide real-time radiation treatment independent of tumor motion, it has the potential to be the next logical progression toward increasing precision of radiation treatment. This preliminary investigation demonstrates BgRT’s dosimetric feasibility compared to non-gated VMAT and IMPT. However, further investigation effectively comparing BgRT to other photon and proton treatment modalities to learn if BgRT has the potential to reduce the dose to adjacent critical structures to a greater extent is needed. Given the necessity for daily tracer injections, BgRT is currently suited toward hypofractionated radiation. However, as this technology enters into the clinic, novel applications of BgRT into the classical 2-Gy per fraction course require exploration, potentially as a boost. Going forward, entirely new fractionation schedules could be developed specifically to exploit the benefits of BgRT.

## Data availability statement

The data analyzed in this study is subject to the following licenses/restrictions: They are the CT Dicom datasets, which we don’t have the privilege to share. Requests to access these datasets should be directed to ooderinde@reflexion.com.

## Author contributions

SS, RB, JW, and SM conceptualized the work. PO, OM, LT, and YV supervised the work. SS and RB collected the data, carried out the treatment planning, and analyzed the data. SS, RB, JW, OO, SM, PO, OM, LT, JC, CW, PB, and YV wrote and revised the manuscript. All authors contributed to the article and approved the submitted version.

## Conflict of interest

JW is a member of the board of advisors and has equity at RefleXion Medical. SM is the founder and CTO of RefleXion Medical. YV is a software architect, RB is a medical physicist, PO is a PET engineer at RefleXion Medical and OO is a clinical scientist at RefleXion Medical.

The remaining authors declare that the research was conducted in the absence of any commercial or financial relationships that could be construed as a potential conflict of interest.

## Publisher’s note

All claims expressed in this article are solely those of the authors and do not necessarily represent those of their affiliated organizations, or those of the publisher, the editors and the reviewers. Any product that may be evaluated in this article, or claim that may be made by its manufacturer, is not guaranteed or endorsed by the publisher.
